# Impact of alloy fluctuations and Coulomb effects on the electronic and optical properties of *c*-plane GaN/AlGaN quantum wells

**DOI:** 10.1038/s41598-019-53693-2

**Published:** 2019-12-11

**Authors:** A. A. Roble, S. K. Patra, F. Massabuau, M. Frentrup, M. A. Leontiadou, P. Dawson, M. J. Kappers, R. A. Oliver, D. M. Graham, S. Schulz

**Affiliations:** 1 Department of Physics and Astronomy, and Photon Science Institute, The University of Manchester, Manchester, M13 9PL United Kingdom; 2 Tyndall National Institute, University College Cork, Cork, T12 R5CP Ireland; 3 Department of Electrical Engineering, University College Cork, Cork, T12 YN60 Ireland; 4 Department of Materials Science and Metallurgy, 27 Charles Babbage Road, University of Cambridge, Cambridge, CB3 0FS United Kingdom; 5 Present Address: School of Science, Engineering and Environment, University of Salford, Salford, Greater Manchester, M5 4WT United Kingdom

**Keywords:** Condensed-matter physics, Materials for devices, Materials for optics, Nanoscale materials, Theory and computation, Nanoscience and technology, Optics and photonics, Physics

## Abstract

We report on a combined theoretical and experimental study of the impact of alloy fluctuations and Coulomb effects on the electronic and optical properties of $c$-plane GaN/AlGaN multi-quantum well systems. The presence of carrier localization effects in this system was demonstrated by experimental observations, such as the “S-shape” temperature dependence of the photoluminescence (PL) peak energy, and non-exponential PL decay curves that varied across the PL spectra at 10 K. A three-dimensional modified continuum model, coupled with a self-consistent Hartree scheme, was employed to gain insight into the electronic and optical properties of the experimentally studied $c$-plane GaN/AlGaN quantum wells. This model confirmed the existence of strong hole localization arising from the combined effects of the built-in polarization field along the growth direction and the alloy fluctuations at the quantum well/barrier interface. However, for electrons these localization effects are less pronounced in comparison to the holes. Furthermore, our calculations show that the attractive Coulomb interaction between electron and hole results in exciton localization. This behavior is in contrast to the picture of independently localized electrons and holes, often used to explain the radiative recombination process in $c$-plane InGaN/GaN quantum well systems.

## Introduction

Ultraviolet (UV) light emitting diodes (LEDs) have attracted significant interest for a variety of applications in such diverse areas as sensing (e.g. gases, multiresistent germs), security (e.g. ID cards, banknotes), medical (e.g. blood gas analysis), plant lighting (e.g. “functional food”) or 3D printing (e.g. rapid prototyping)^1–8^. However, to be able to address this wide range of applications, UV LEDs must cover the UVC (280–200 nm), UVB (320–280 nm) and UVA (400–320 nm) wavelength regimes. For this reason the semiconductor alloy aluminium gallium nitride (AlGaN) has been used in state-of-the-art UV LEDs, since it allows in principle to tailor the emission wavelength of these devices through changing the Al and Ga content.

Despite the huge potential of this material system, fundamental aspects of the radiative and non-radiative recombination processes in GaN/AlGaN quantum wells (QWs), forming the heart of the active region of UV LEDs, are still not well understood. For instance, metal-organic vapor deposition growth of wurtzite $c$-plane GaN/AlGaN-based QWs, utilizing sapphire substrates, leads to defect densities in the range of $1{0}^{8}$ cm^−2^ ^9^. With these high defect densities it is remarkable that GaN/AlGaN devices function at all. In general, the widely accepted explanation for the defect insensitivity of heterostructures based on wurtzite III-N materials, such as AlGaN, InGaN or even AlInN systems, is that localization effects prevent the carriers from reaching these defects^10–12^. Significant experimental and theoretical efforts have been made to understand the nature of carrier localization in InGaN/GaN QW systems and its impact on the QW optical properties^13–16^. In comparison to InGaN, far less attention has been directed towards these phenomena in $c$-plane GaN/AlGaN QWs, especially in the theoretical analysis of these systems. Carrier localization effects and their potential impact on the nature of the radiative recombination process have been widely neglected, despite experimental evidence of localization phenomena^17,18^. One clear indicator is the so-called “S-shape” dependence of the photoluminescence (PL) peak energy with temperature. This anomalous temperature behavior of the PL peak energy has been observed in both $c$-plane InGaN/GaN^9,19– 23^ and GaN/AlGaN QWs^17,18,24,25^. However, it is important to note that while these two systems reveal the same behavior, fundamental differences exist between InGaN/GaN and GaN/AlGaN wells. While in InGaN/GaN heterostructures in general the overall polarization vector field is dominated by piezoelectric effects, in GaN/AlGaN systems the spontaneous polarization is comparable, and might even be larger than the piezoelectric response^26^. Further differences between the material systems arise from differences in the valence and conduction band offset values and their compositional dependence^27–29^ as well as the electron and hole effective masses^30^. In InGaN systems the explanation for the “S-shape” temperature dependence of the PL peak energy is based on a thermal redistribution of carriers between localized states, which originate from (random) alloy fluctuations in the well. However, in $c$-plane GaN/AlGaN QWs, where the well is made of the *binary* compound GaN, the active region does not contain any alloy fluctuations and only in the barrier material do such alloy fluctuations exist. This gives rise to the question of the nature of carrier localization in $c$-plane GaN/AlGaN QWs. Previous studies on different material systems, such as zincblende GaAs/AlGaAs QWs, suggest that well width fluctuations and/or random alloy fluctuations in the AlGaAs barrier^31^, or more precisely at the well/barrier interface, can lead to carrier localization effects. Building on this finding, one could expect that these effects are even more pronounced in wurtzite $c$-plane GaN/AlGaN QWs, given that the growth along the polar $c$-axis results in strong electrostatic built-in fields in this direction. These fields, which are not present in zincblende GaAs/AlGaAs QWs grown along the [001]-direction, are due to the combination of the underlying wurtzite crystal structure of the GaN and AlGaN systems and the growth of the heterostructure along the crystallographic $c$-axis. In such a case, and when growing QW structures, both spontaneous and piezoelectric polarization vector fields oriented along the cyrstallographic $c$-axis in III-N materials arise in general. Given that the polarization fields are different in GaN and AlGaN, for instance, in a heterostructure one is left with a discontinuity in these fields. As a consequence, III-N nanostructures exhibit very strong electrostatic built-in fields of the order of MV/cm. In $c$-plane GaN/AlGaN QWs, the electrostatic built-in fields lead to the situation that electron and hole wave functions are displaced towards the GaN/AlGaN interfaces. However, in comparison to $c-$plane InGaN/GaN QW systems, theoretical calculations that account for or discuss alloy fluctuations in GaN/AlGaN QWs explicitly, are sparse^17,18,32^. Most often their electronic and optical properties are treated in the framework of one-dimensional continuum-based models, which account for strain and built-in fields, treating the AlGaN alloy as an effective medium that can be described by averaged parameters^33^. In such works, effects of alloy fluctuations are only accounted for by a (non-linear) compositional dependence of the involved material parameters. Furthermore, when going beyond the single-particle picture, excitonic effects are introduced in a second step with similar approximations, namely considering only a one-dimensional system and an average material parameter description for the well and barrier material.

In this paper we address the question of carrier localization effects and the connected question of the nature of the radiative recombination process in $c$-plane GaN/AlGaN QWs. To do so, we have studied these systems in the framework of a combined experimental and theoretical approach, allowing us to refine the model with experimental input. We have investigated two $c$-plane GaN/AlGaN multi-QW (MQW) samples which were grown by metal-organic chemical vapor deposition. These two samples differ in well width: Sample A has wells with a width (${L}_{w}$) of $2.4$ nm, and Sample B has wells with a much larger width of $6.8$ nm. This allows us to study the impact of the well width on changes in the optical properties of $c$-plane GaN/AlGaN QWs and how carrier localization effects contribute. An important aspect for our investigations is to connect the optical properties to the structural properties of the system. Therefore, the structural characteristics of the MQWs have been analyzed by transmission electron microscopy (TEM) and X-ray diffraction (XRD) measurements. The information gained has been included in the theoretical modelling of the electronic and optical properties of these structures in the frame of a three-dimensional modified continuum-based model, which accounts explicitly for alloy fluctuations in the barrier and resulting carrier localization. This is different from commonly used theoretical models in the literature on GaN/AlGaN QWs, where the effects of alloy fluctuations in the barrier are explored by a non-linear compositional dependence of averaged material parameters. To gain insight into any excitonic effects, self-consistent Hartree calculations have been performed. The self-consistent approach captures effects such as charge density re-distribution due to the attractive Coulomb interaction between electron and hole. This important contribution has been neglected in previous studies using a fully three-dimensional theoretical description of electronic and optical properties of $c$-plane GaN/AlGaN QWs. Finally, the optical properties of the samples have been investigated and analyzed by temperature dependent PL spectroscopy and PL decay time measurements at 10 K. These measurements provided information on carrier localization effects, which are attributed to the well/barrier interface roughness introduced by random alloy fluctuations in the barrier, while also shedding light on radiative recombination processes in these systems.

## Results and Discussion

In this section we present the results of our combined experimental and theoretical analysis of the electronic and optical properties of $c$-plane GaN/AlGaN MQWs. In a first step we give an overview of the structural properties of the samples. In a second step, we present our experimental analysis of the optical properties of these systems. Finally, building on the available structural information on the different samples, the results of our theoretical investigation on their electronic and optical properties are discussed and connected to the experimentally observed optical features.

### Structural analysis

**Figure 1:**
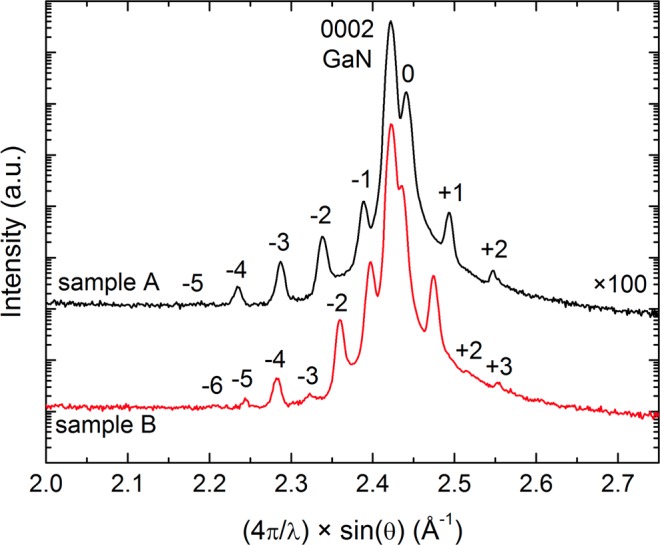
XRD $\omega$-$2\theta$-scans of the 0002 reflection showing the satellite peaks of the GaN/AlGaN MQW structures of Sample A (top) and Sample B (bottom).

High resolution XRD $\omega$-$2\theta$-scans of the 0002 reflection are shown in Fig. 1 for the Samples A (top) and B (bottom). The intense sharp peak in the center of the scan at $\sim 2.42$ Å${}^{-1}$ originates from the GaN buffer layer, while the adjacent peak on its right shoulder is the zero-order peak of the GaN/AlGaN MQW structure. Higher order satellite peaks can be observed on either side of the zero-order MQW peak. Their spacing in reciprocal space is slightly larger for Sample A than for Sample B, revealing the larger average GaN/AlGaN period thickness in Sample B. The occurrence of missing satellite peaks indicates the absence of gross well fluctuations and provides evidence for the high structural uniformity in the GaN/AlGaN MQW structures. Although the number of observable satellite peaks is somewhat limited by the low scattering factor of the AlGaN barriers, their position and relative intensity could be used for comparisons with simulated intensity profiles. These revealed that Sample A consisted of ${L}_{w}$ = (2.4 $\pm$ 0.1) nm wide GaN wells and ${L}_{b}$ = (9.8 $\pm$ 0.1) nm thick Al${}_{x}$Ga${}_{1-x}$N barriers with an average Al-content $x$ of (19.0 $\pm$ 0.3)%. Sample B had ${L}_{w}\,=\,$(6.8 $\pm$ 0.1) nm wide GaN wells and ${L}_{b}$ = (9.5 $\pm$ 0.1) nm thick Al${}_{x}$Ga${}_{1-x}$N barriers with an Al-content of (18.2 $\pm$ 0.3)%.

**Figure 2:**
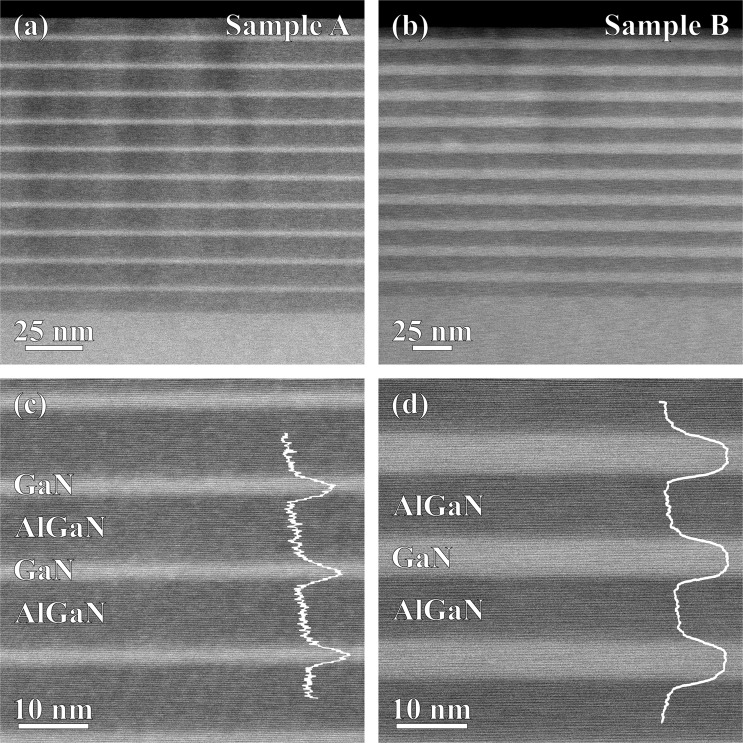
HAADF-STEM images of QW stack in (**a**,**c**) Sample A and (**b**,**d**) Sample B observed along the $< 11\bar{2}0 >$ zone axis. In (**c**,**d**), profile of the HAADF-STEM intensity (a.u.) is plotted to highlight that the bottom interface of the QW is sharper than the top interface (the profiles have been smoothened to attenuate the impact of lattice fringes).

Cross-sectional imaging of the QW stack was performed by high angle annular dark field scanning transmission electron microscopy (HAADF-STEM), as illustrated in Fig. 2. HAADF-STEM provides Z-contrast, hence in these images the GaN QWs appear brighter than the AlGaN barriers. The QW thickness measured from TEM agrees well with those obtained by XRD, with ${L}_{w}$ = (2.3 $\pm$ 0.2) nm and ${L}_{w}$ = (6.6 $\pm$ 0.2) nm for Sample A and B, respectively. Figure 2 shows no noticeable sign of QW thickness fluctuation throughout the QW stack – *i.e*. no topological roughness – in both samples. However, we note that the HAADF-STEM intensity profile taken across the QW is slightly asymmetric in both samples (cf. Fig. 2(c,d)). In comparison to the bottom well/barrier interface, the top well/barrier interface appears to be unintentionally graded in composition. Similar grading of the top well/barrier interface has been observed in InGaN/GaN QWs^34^. However, the interfaces observed here are rather sharper than are typically observed in InGaN/GaN QWs^35^. In InGaN QWs, this grading is attributed to the segregation of indium at the QW surface, which is then incorporated during the growth of the barriers. Here, a similar surface accumulation of Ga may occur.

### Experimental analysis of the optical properties

**Figure 3:**
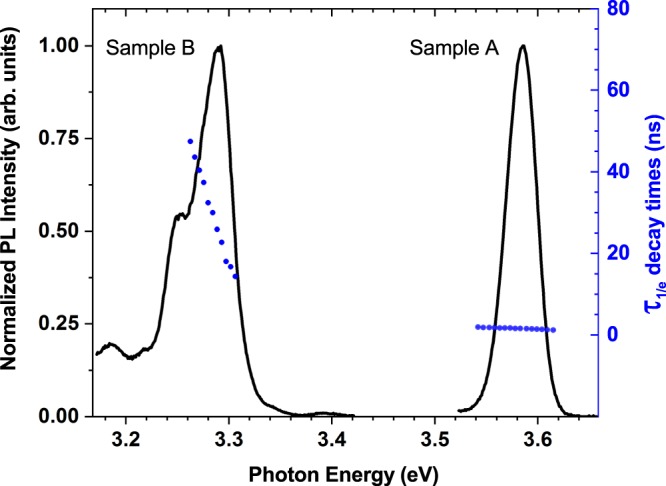
Normalized PL spectra and $1/e$ decay times obtained at a temperature $T=10$ K for GaN/Al${}_{0.18}$Ga${}_{0.82}$N MQWs with well widths ${L}_{w}=2.4$ nm (Sample A, right) and ${L}_{w}=6.8$ nm (Sample B, left). The solid curves correspond to PL spectra obtained with a photo-excitation energy of $4.66$ eV ($\lambda =266$ nm). The circles represent the ${\tau }_{1/e}$ PL decay times measured across the displayed PL spectra.

Having discussed the structural properties of the two $c$-plane GaN/AlGaN MQW systems, we now turn to their optical properties using a range of different techniques. PL spectroscopy, temperature dependent PL spectroscopy and PL decay time measurements have been performed to gain insight into the fundamental properties of the two samples. More details about the measurements are given in the “Methods” section. We start our analysis here with the results from low temperature PL measurements. Figure 3 depicts the low temperature ($T=10$ K) PL spectra of Sample A (${L}_{w}=2.4$ nm) and B (${L}_{w}=6.8$ nm) for a photo-excitation energy of $4.66$ eV (see “Methods") corresponding to an excitation above both the GaN and Al${}_{0.18}$Ga${}_{0.82}$N band gaps of $3.45$ eV and $3.91$ eV, respectively. Given that the decays were found to be non-exponential, we give the decay time ${\tau }_{1/e}$ as the time taken by the PL signal to fall to $1/e$ of its maximal value. These decay times (circles) are shown across the PL spectra for both samples. Before looking at ${\tau }_{1/e}$ in detail, we start here with the discussion of the PL spectra. Sample A (${L}_{w}=2.4$ nm) shows a peak PL energy of $3.59$ eV with a full-width at half maximum (FWHM) of $35$ meV. For Sample B (${L}_{w}=6.8$ nm), the PL spectrum is red-shifted with respect to that of Sample A due to the larger well width ${L}_{w}$. Here, we find a PL peak energy of 3.29 eV and a FWHM of 60 meV . The PL peak that can be seen on the spectrum of sample B at $3.25$ eV is attributed to the well known Donor-Acceptor-Pair (DAP) recombination at 3.257 eV^36,37^. This is confirmed by the fact that this peak quenches rapidly with temperature, which is consistent with the behavior reported for DAP recombination features in GaN^36,38– 40^. Turning to the FWHM values, the above measured large values give a first indication of carrier localization effects in our $c$-plane GaN/AlGaN MQW samples. In $c$-plane InGaN/GaN QWs similar effects have been observed and attributed to carrier localization, which result in variations in electron and hole ground state energies depending on the local alloy configurations and fluctuations in the confining QW width (well width fluctuations)^21,41^. Due to these fluctuations, a broad PL spectra is expected and observed. To further investigate potential carrier localization effects in GaN/AlGaN MQWs, PL spectra as a function of the temperature $T$ have been studied. In these measurements an excitation energy of 3.815 eV was used and the PL peak was tracked over a temperature range of $T=10$ to $T=300$ K. It should be noted that the PL peak for Sample B coincides with a number of peaks attributed to one of the following: deep-acceptor related luminescence (the so-called blue band), recombination at DAPs or even structural defects in GaN^37^. As a consequence, it was difficult to track the QW emission peak at temperatures above $T=180$ K. Thus, for Sample B we focus on the temperature range $T=10$ K to $T=180$ K.

**Figure 4:**
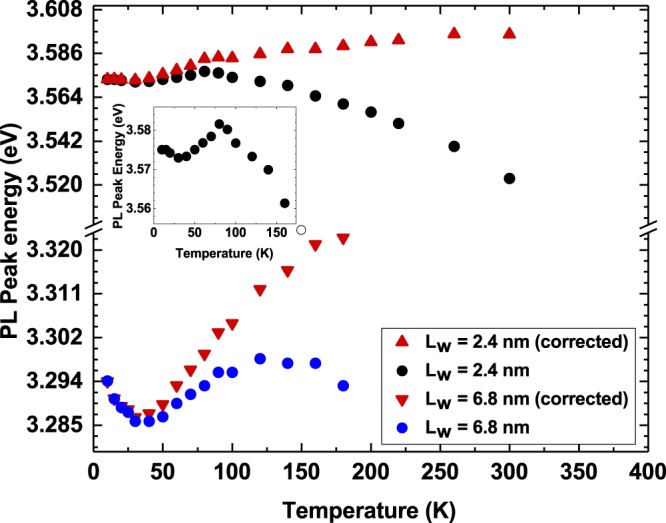
Peak PL energy as a function of temperature $T$ for GaN/Al${}_{0.18}$Ga${}_{0.82}$N MQWs with well widths ${L}_{w}$ of ${L}_{w}=2.4$ nm (Sample A) and ${L}_{w}=6.8$ nm (Sample B). The filled circles show the raw data, and the triangles show the data with the band gap temperature dependence removed using the two-parameter Varshni expression. The inset shows the PL peak energy data for Sample A, for $T\le 160$ K.

The results of this investigation are displayed in Fig. 4. Both samples exhibit an anomalous behavior (“S-shape”) of the PL peak energy (filled circles) as a function of temperature $T$. Initially, the PL peak position red-shifts from $T=10$ K to $T=30$ K, blue-shifts from $T=30$ K to $T=150$ K and then red-shifts following the temperature-dependent energy gap shrinkage. The empirical two-parameter Varshni expression ${E}_{g}(T)={E}_{g}(0)-\alpha {T}^{2}/(\beta +T)$ was used to remove the temperature dependence of the band gap, using the Varshni parameters $\alpha =1.235\times 1{0}^{-3}$eV/K and $\beta =1435$ K^42,43^. The corrected data is shown in Fig. 4 by the red triangles. The inset in Fig. 4 shows a close-up view of the data for Sample A ({L}_{w} = 2.4 nm) up to $T=160$ K. As already discussed above, this “S-shape” temperature dependence of the peak PL energy is a spectral signature of carrier localization and is explained in InGaN/GaN MQWs and other disordered systems by a thermal redistribution of the carriers between localized states. Bell *et al.*^44^ described a model according to which, starting from a random distribution of carriers among the localization sites at low temperatures (the so-called “freeze-out regime”) and following an increase in temperature, the carriers can thermalize out of shallow states and drop to deeper potential wells, resulting in a red shift of the peak PL energy. In $c$-plane InGaN/GaN wells, these localized states originate from a combination of random alloy fluctuations, electrostatic built-in fields and well-width fluctuations. With the temperature increasing further the carriers “hop out” of the deep localization states and once more occupy shallow localization states, as shown by a blue shift of the peak PL energy. As the temperature keeps increasing, the red-shifting PL peak energy reflects the phonon-mediated energy gap shrinkage becoming the dominant effect. The observed “S-shape” temperature dependence of the PL peak position in our GaN/Al${}_{0.18}$Ga${}_{0.82}$N MQWs is consistent with the results obtained by other groups on such heterostructures^45,46^.

To gain insight into the question of how carrier localization might change with well width in $c$-plane GaN/Al${}_{0.18}$Ga${}_{0.82}$N QWs, we calculated the maximum energy difference, ${\Delta }_{\,PL}^{Varshni\,}$, between the lowest PL peak energy and the maximum of the PL peak energy with the temperature dependence removed using Varshni’s formula for the two samples studied. For Sample A (${L}_{w}=2.4$ nm) we find a shift of ${\Delta }_{\,PL}^{Varshni\,}$=26 meV between $T=30$ K and $T=300$ K. For Sample B (${L}_{w}=6.8$ nm) this shift amounts to ${\Delta }_{\,PL}^{Varshni\,}$ = 35 meV between $T=30$ K and $T=180$ K. The higher value observed in case of the MQW with ${L}_{w}=6.8$ nm accompanied by the more distinct “S-shape” (cf. Fig. 4) indicates that the impact of carrier localization effects on the optical properties tends to increase with increasing well width. We will come back to this point further below when discussing the results of our theoretical studies.

**Figure 5:**
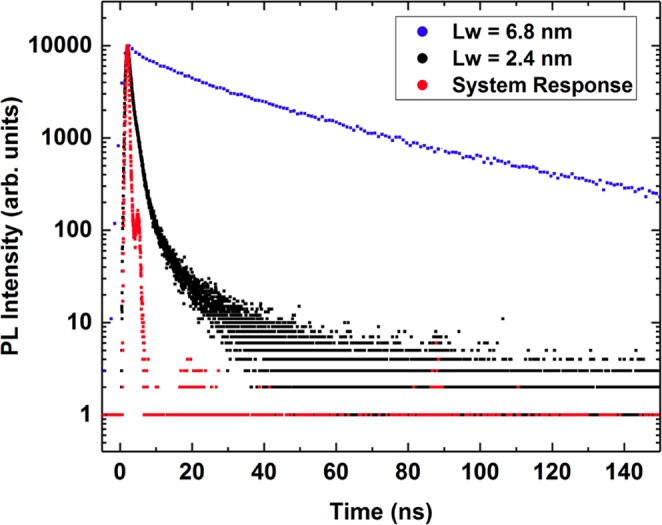
PL time decay curves measured at the PL peak energy of the $T=10$ K spectra for GaN/Al${}_{0.18}$Ga${}_{0.82}$N MQWs with well widths ${L}_{w}$ of 2.4 nm (Sample A) and 6.8 nm (Sample B) together with the system response of the PL decay time measuring setup.

While the temperature dependent PL data gives a clear indication of carrier localization effects in $c$-plane GaN/AlGaN QWs, it does not give any insight into the nature of the recombination process. To shed light onto this question, we have performed PL time decay measurements. The results of this analysis are shown in Fig. 5. Overall, we find here that the decay transients are non-exponential. This feature is also observed in $c$-plane InGaN/GaN QWs, where the widely used explanation for this behavior is that electrons and holes are “independently” localized, leading to a statistical variation in the electron and hole wave function overlap and therefore to a non-exponential decay transient^13,47^. This also results in the situation that the radiative lifetime, which is dependent on the electron-hole overlap integral, varies across the low temperature PL spectrum. We have extracted the radiative lifetime ${\tau }_{1/e}$ across the PL spectrum from the PL time decay measurements with the photo-excitation energy of $4.66$ eV as shown in Fig. 3. As the decays are non-exponential, we have determined the decay time by the time taken for the photon count to decrease by a factor of 1/e. The 1/e decay times for Sample B show that ${\tau }_{1/e}$ varies across the spectrum, comparable to the recombination energy dependence of the radiative lifetime reported in both InGaN/GaN^13,48–50^ and GaN/AlGaN QWs^46,51^ and attributed to carrier localization. Here, the decay times increase from 14 ns to 40 ns when the detection photon energy decreases from 3.30 eV to 3.27 eV. However, Sample A reveals a noticeably different behavior, with much faster radiative decay times which are approximately constant across the spectrum, increasing only slightly from 1.42 ns at 3.60 eV to 1.70 ns at 3.57 eV. Our results indicate that in $c$-plane GaN/AlGaN QWs the well width has a noticeable impact on the spectral dependence of the radiative lifetime and could indicate a change in the radiative recombination process.

Having discussed our experimental studies on GaN/AlGaN QWs, we turn now and focus on the electronic and optical properties of these systems from a theoretical perspective. Building on our experimental findings, special attention is paid to potential carrier localization effects and consequence for the radiative lifetime. This is presented in the following section.

### Theoretical analysis of the electronic and optical properties

To calculate the electronic and optical properties of the $c$-plane GaN/AlGaN QWs, a fully three-dimensional, modified, continuum-based ${\bf{k}}\cdot {\bf{p}}$ model has been employed here. This allows us to treat alloy fluctuations in the barrier material explicitly. Overall, the approach is similar to the one used by Watson-Parris *et al*.^21^ to study the electronic and optical properties of $c$-plane InGaN/GaN QWs. However, our model here is coupled to a self-consistent Hartree scheme, therefore also accounting for Coulomb effects (excitonic effects). More details about the theoretical framework are given in the “Methods” section.

We have investigated here the two $c$-plane GaN/AlGaN MQW structures. In the first system the well width is ${L}_{w}$ = 2.5 nm, which is in good agreement with the well width of Sample A ${L}_{w}=(2.4\pm 0.1)$ nm. For the second system we have assumed a well width of ${L}_{w}=6.75$ nm, thus in good agreement with Sample B ${L}_{w}=(6.8\pm 0.1)$ nm. The barrier width in both MQW systems is 10 nm and the Al content in the barrier is 18%, building on the experimental findings given above. For the AlGaN barrier a random alloy situation was assumed. To sample different random alloy configurations in the barrier, the calculations have been repeated 20 times for each of the two MQW structures. In doing so, we can analyze the impact of different random alloy configurations on the electronic and optical properties of the two MQW systems. For the main theoretical results presented here, we do not explicitly include well width fluctuations, since there is no experimental indication for these features (see the “Structural Analysis" section). Thus, the only interface roughness included originates from alloy fluctuations at the well/barrier interface. This is in contrast to $c$-plane InGaN/GaN QWs, where well width fluctuations have been observed and included in theoretical investigations as disk-like objects at the interface between InGaN QW and GaN barrier^21,41^. However, to study the potential impact of such structural inhomogeneities on the electronic and optical properties of GaN/AlGaN systems, we have performed additional calculations where disk-like well width fluctuations, similar to $c$-plane InGaN/GaN QW systems, have been taken into account. Results of these calculations will be discussed briefly below.

**Figure 6:**
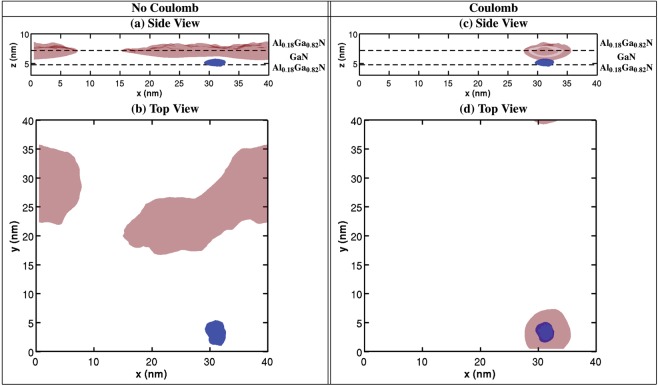
Isosurface plots of the electron (red) and hole (blue) ground state charge densities in the absence (left column, (**a**,**b**)) and in the presence of (right column, (**c**,**d**)) Coulomb (excitonic) effects. The results are displayed for an arbitrarily chosen alloy configuration representing Sample A. The isosurface corresponds to 25% of the respective maximum charge density values. The well boundaries are schematically indicated by dashed lines.

Our framework has now been used to investigate the electronic and optical properties of Sample A and B from a theoretical perspective. In a first step the calculations have been performed in the absence of Coulomb (excitonic) effects. Figure 6(a,b) depict the electron (red) and hole (blue) ground state charge densities of the $c$-plane QW system of Sample A for an arbitrarily chosen microscopic configuration. The charge densities are displayed at 25% of their respective maximum values and are shown from two different viewpoints, (a) perpendicular (Side View) and (b) parallel (Top View) to the wurtzite $c$-axis ($z$-axis). Several features of the charge densities are now of interest. First of all, the carriers are spatially separated along the $c$-axis (growth direction) due to the presence of the strong electrostatic built-in field along this direction. This results in the situation that electrons and holes are localized at the well/barrier interface and are thus susceptible to probe any potential fluctuations in this region. Figure 6(a,b) also reveal strong hole wave function localization effects induced by the effect that the built-in field forces the carriers to the well/barrier interface and therefore being exposed to the interface roughness due to the random alloy fluctuations in this region. In terms of the hole wave function localization characteristics, we find here a behavior that is similar to $c$-plane InGaN/GaN QWs^13,41^. The ground state electron charge density exhibits a much more delocalized character, at least when compared to the hole. The observations made above for the arbitrarily chosen configuration hold for all other 19 alloy configurations studied here.

In general, our results on GaN/AlGaN systems are consistent with the earlier studies by Gallart *et al*.^18^ and Rigutti *et al*.^17^ in terms of the electron and hole localization characteristics in $c$-plane GaN/AlGaN QWs. Also, the observed carrier localization effects are consistent with the experimentally observed “S-shape” temperature dependence of the peak PL energies, which requires the presence of localized states in the system. Our calculations reveal similar results for the ${L}_{w}=6.8$ nm well in terms of the electron and hole ground state localization features. But, our experimental studies on the temperature dependence of the PL peak energy indicated that carrier localization effects tend to be more pronounced in the wider well (Sample B) when compared to the narrower well (Sample A). This argument was based on the observation that the “S-shape” temperature dependence of the peak PL energy in case of Sample B is more pronounced in comparison to Sample A. To gain initial insight from theory into the question how carrier localization features might change with well width, additional calculations have been performed in which the QW systems are treated in a virtual crystal approximation (VCA), meaning that the barrier is described as an effective material with averaged material parameters. Thus no interface roughness due to locally varying Al and Ga content is accounted for. More details on the VCA calculations are given in “Methods” below. Similarly to the experiment where the Varshni formula and the measured peak PL energies have been used to gain insight into the impact of the well width on carrier localization, we have calculated the energy difference ${\Delta }_{\,GS}^{VCA \mbox{-} Alloy\,}$ between the ground state transition energy obtained from the VCA calculation and the average transition energy resulting from our model that accounts for local variations in Al and Ga content. It is important to note that this analysis can only provide qualitative insight since a quantitative analysis would require a thorough benchmark of the VCA against given experimental data, *e.g*. reproducing the energy gap of the system at low temperatures. However, even without adjusting the VCA by introducing for instance bowing parameters, using the same VCA for Sample A and B allows for a consistent comparison of the impact of the alloy fluctuations on the electronic structure of the two systems, bearing in mind that the main difference in Sample A and B in terms of its structural properties is the well width and not the Al content. From theory we find for Sample A a value of ${\Delta }_{\,GS}^{VCA \mbox{-} Alloy}=23$ meV. For Sample B the value for ${\Delta }_{\,GS}^{VCA \mbox{-} Alloy\,}$ is approximately three times larger than for Sample A. This indicates a much more pronounced effect of the interface roughness on the electronic and optical properties in the wider well. This increase in ${\Delta }_{\,GS}^{VCA \mbox{-} Alloy\,}$ is consistent with the much more pronounced “S-shape” of Sample B (${L}_{w}=6.8$ nm) when compared with Sample A (${L}_{w}=2.4$ nm) as one can infer from Fig. 4. We attribute this effect to the larger potential difference between the upper and lower interface of the QW for Sample B in comparison to Sample A and thus to a stronger localization of the wave functions at the well/barrier interface. We come back to this aspect further below.

In a second step, we now study the impact of Coulomb (excitonic) effects on the results. Coulomb effects have been included in the work by Rigutti *et al*.^17^ as an energetic shift to the single-particle energy gap (energy difference between the lowest electron and highest valence state). However, such an approach does not account for potential charge density re-distributions, which might originate from the attractive interaction between electron and hole. Previous theoretical studies on $c$-plane InGaN/GaN QWs have revealed that strong electron and hole localization effects dominate over the attractive Coulomb interaction between these carriers at low temperatures^41,52^. Therefore, in $c$-plane InGaN/GaN QWs the single-particle description of the electron and hole ground state charge densities represent a reasonable description of the excitonic charge densities at low temperatures. The Coulomb effect mainly results in a red-shift of the single-particle transition energy^41,52^. As already discussed above, in $c$-plane InGaN/GaN QWs one is therefore left with a system that can be approximated by independently localized carriers. But, it is important to note that well width fluctuations, in addition to (random) alloy fluctuations, introduce localization centers for electrons in $c$-plane InGaN/GaN QWs. In the studied GaN/Al${}_{0.18}$Ga${}_{0.82}$N QWs, well width fluctuations of the dimensions observed in InGaN/GaN QWs are absent, as discussed above. Here, in the single-particle picture and consistent with Rigutti *et al*.^17^, the electron charge density does not exhibit strong localization characteristics, at least not on the scale of the hole. Therefore, it remains to be seen how excitonic effects affect electron and hole states. The self-consistent Hartree approach we apply captures potential charge density re-distributions and can thus throw light on the question if the recombination process is similar to $c$-plane InGaN/GaN QWs with independently localized carriers or if it is different. Figure 6(c,d) depict the electron and hole ground state charge densities in the presence of Coulomb (exciton) effects. The isosurfaces of electron (red) and hole (blue) charge densities are again displayed at 25% of their respective maximum values and for the two different viewpoints. We observe here that the hole wave function is still strongly localized, even at the same position as in the absence of Coulomb effects (cf. Fig. 6(b)). For the electron charge density the situation drastically changes. Here, we observe that the electron localizes about the hole. Thus, the in-plane separation in the single-particle picture (cf. Fig. 6(a,b)) is compensated by the attractive Coulomb interaction between electron and hole (cf. Fig. 6(c,d)). However, the Coulomb effect cannot overcome the spatial electron and hole wave function separation along the $c$-axis, stemming from the intrinsic electrostatic built-in field along this direction. This behavior is found for all 20 different microscopic configurations studied. Therefore, our fully self-consistent three-dimensional calculation reveals that in contrast to $c$-plane InGaN/GaN QW systems, electrons and holes are *not* independently localized in $c$-plane GaN/AlGaN QW systems, at least not for the Al contents studied here. We observe the same behavior for the ${L}_{w}=6.75$ nm well system, indicating that independent of well width the investigated structures show exciton localization effects. Furthermore, our calculations including well width fluctuations (not shown here) reveal the same behavior, meaning that electron and hole localize in the same spatial in-plane position. All this therefore shows that the inclusion of Coulomb effects for these systems is important to describe their electronic and optical properties.

**Figure 7:**
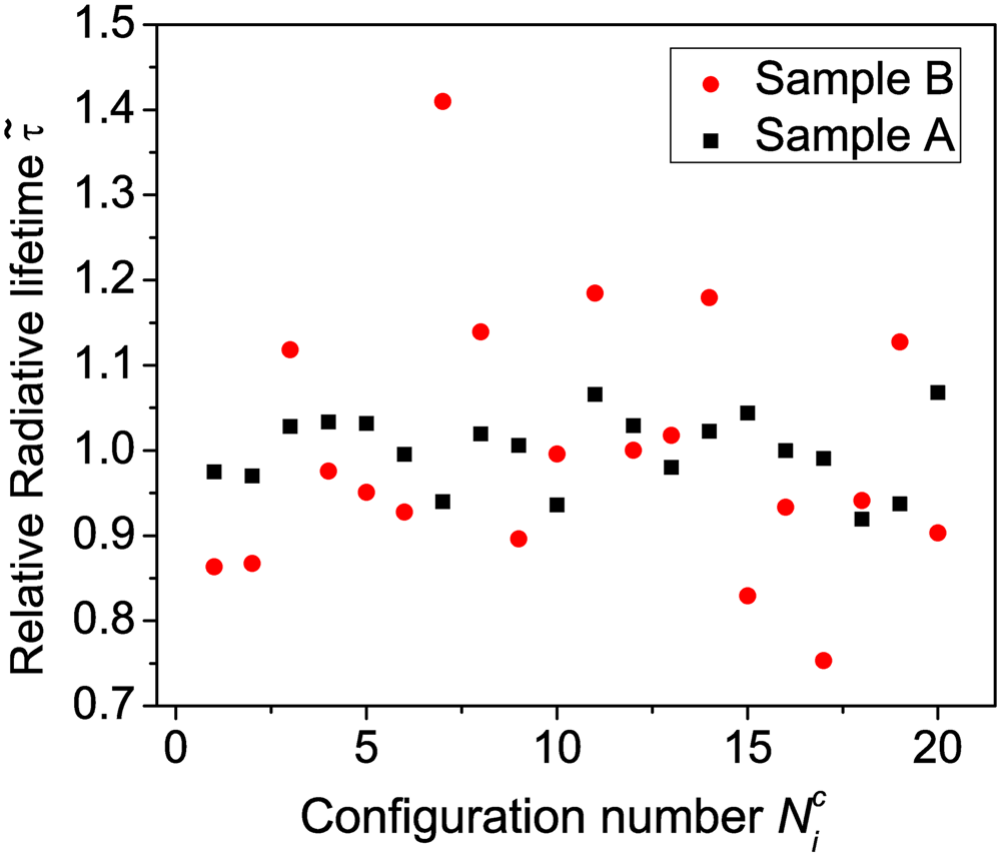
Calculated relative radiative lifetime $\widetilde{\tau }$ as a function of alloy configuration number ${N}_{i}^{C}$. The results for the narrower well system (Sample A) are given by the black squares; data for the wider well system (Sample B) are denoted by the red circles. More information is given in the text.

**Figure 8:**
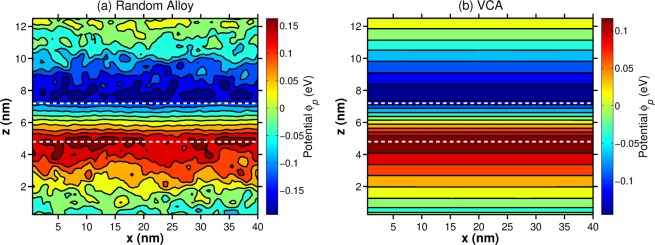
Contour plot of built-in potential ${\phi }_{p}$ of $c$-plane Al${}_{0.18}$Ga${}_{0.72}$N/GaN QW system representing Sample A for a slice through the the $x-z$-plane. The $z$-axis is parallel to the wurtzite $c$-axis. The white dashed lines schematically indicate the QW interfaces. (**a**) Random alloy treatment (arbitrarily chosen microscopic configuration) and (**b**) result from a virtual crystal approximation (VCA).

The question now remains how this picture of exciton localization fits with the observed non-exponential decay transients and that the variation of the radiative lifetime $\tau$ across the PL spectrum seems to be well width dependent. To shed light onto these aspects, we have calculated the radiative lifetime ${\tau }^{theo}$ for each of the twenty different microscopic configurations for the two different MQW systems. Our experimental studies discussed above reveal that for the narrower well width system (Sample A), $\tau$ is almost constant across the PL spectrum while for Sample B $\tau$ varies significantly. To address this finding from a theoretical perspective and to compare the calculated change in radiative lifetime between the narrow and the wide well in general, we study here the *relative/normalized* radiative lifetime $\widetilde{\tau }({N}_{i}^{C})$, defined by $\widetilde{\tau }({N}_{i}^{C})={\tau }^{theo}({N}_{i}^{C})/{\tau }_{\,average}^{theo\,}$, as a function of the configuration number ${N}_{i}^{C}$. Here, ${\tau }^{theo}({N}_{i}^{C})$ is the calculated radiative lifetime for configuration ${N}_{i}^{C}$, while ${\tau }_{\,average}^{theo\,}$ denotes the radiative lifetime averaged over all 20 configurations. These calculations have been performed for the two different well width systems and the results are displayed in Fig. 7 for the narrower (Sample A, black squares) and wider (Sample B, red circles) MQWs. In case of Sample A, $\widetilde{\tau }$ varies only slightly with configuration number ${N}_{i}^{C}$ (standard deviation $\sigma =0.04$). This is in contrast to the wider well system (Sample B), where the variation in $\widetilde{\tau }$ is larger with respect to the configuration number ${N}_{i}^{C}$(standard deviation $\sigma =0.15$). This finding is consistent with the experimentally found behavior that $\tau$ is almost constant in Sample A when compared to the results for Sample B. In general, this observation can be explained in the following way. The fluctuations in Al and Ga content at the well/barrier interface lead to significant fluctuations in the built-in potential. An example for this feature is shown in Fig. 8(a), displaying the built-in potential ${\phi }_{p}$ for a slice through the supercell of an arbitrarily chosen configuration of the narrower well system (Sample A). The data is shown in the $x-z$-plane. Here, the $z$-axis is parallel to the wurtzite $c$-axis. For comparison, the built-in potential of the same QW system (same well width) but in VCA is shown in Fig. 8(b). From Fig. 8(a,b) we clearly see the strong local built-in potential fluctuations due to the alloy fluctuations in the barrier material. These fluctuations, especially at the well/barrier interface will now affect carrier localization. Thus, even though our calculations indicate exciton localization effects, variations in the electrostatic built-in field will also (slightly) affect the wave function overlap. Given that the radiative lifetime ${\tau }^{theo}({N}_{i}^{C})$ is inversely proportional to the wave function overlap, ${\tau }^{theo}({N}_{i}^{C})$ should also be affected by changes in the wave function overlap. Two factors contribute now to the situation that the wider well shows a more pronounced variation in the radiative lifetime across the spectrum. First, the built-in potential difference between the top and the bottom interface is larger in the wider well when compared to the narrower one (not shown here). This leads to even stronger electron and hole wave function localization at the well/barrier interfaces, and thus local fluctuations in alloy and built-in fields in this region are expected to contribute more strongly to carrier localization in the wider well when compared to the narrower well. In combination with the wider well width, the wave function overlap between electron and hole is significantly further reduced in the wider well in comparison to the narrower one. The second factor that comes into play is how the radiative lifetime in $c$-plane III-N heterostructures changes with well width. Here, in general an exponential increase of $\tau$ with well width is observed^53^. Thus on an absolute scale, slight changes in the wave function overlap due to alloy induced built-in field variations should have a more pronounced effect on the radiative lifetime in the wider well. Therefore, taking all these findings together one could expect a stronger variation of the radiative lifetime across the PL spectrum in the wider well when compared to the narrower well, even with exciton localization effects. This is confirmed here by both the theoretical calculations as well as the measurements.

It is important to note, that even though our calculations indicate exciton localization effects, our system is different to, for instance, non-polar InGaN/GaN QW systems where exciton localization has also been observed and discussed^54^. This stems from the fact that in the $c$-plane GaN/AlGaN QWs studied here, strong electrostatic built-in fields along the growth direction are present and as discussed above, fluctuations in the local alloy configuration leads to noticeable fluctuations in the built-in field. Consequently, this results in fluctuations in the electron hole wave function overlap, even for a localized exciton in the same in-plane position but with electron and hole wave functions still separated along the growth direction. This macroscopic built-in field is absent in non-polar III-N systems. Due to the absence of the macroscopic field in these non-polar systems, excitonic effects lead to the situation that electron and hole localize in the same spatial position, not only in the growth plane but also out of growth plane. Thus even though we observe exciton localization in both $c$-plane GaN/AlGaN QWs and for instance non-polar InGaN/GaN QWs, the underlying physics of the recombination process is expected to be different due to the presence/absence of the macroscopic built-in field in these two systems.

## Conclusion

We have presented a detailed experimental and theoretical study of the electronic and optical properties of $c$-plane GaN/AlGaN QWs. Our measurements reveal an “S-shape” temperature dependence of the PL peak energies, which is indicative of carrier localization effects in the system. Our calculations, using a three-dimensional modified continuum-based model, which accounts for random alloy fluctuations in the barrier material (AlGaN), reveal strong hole localization effects originating from the combined effects of built-in field and alloy fluctuations at the well/barrier interface in $c$-plane GaN/AlGaN QWs. Thus, we show here that already alloy fluctuations at the well/barrier interface are sufficient in $c$-plane III-N QW systems to bring about strong hole localization effects. The situation observed here is slightly different to $c$-plane InGaN/GaN systems, where in addition to strong hole localization effects electrons are also significantly localized by well width fluctuations.

Furthermore, our PL time decay measurements reveal non-exponential decay curves, which in $c$-plane InGaN/GaN QW systems have been regarded as an indicator for independently localized electron and hole wave functions. Similar to $c$-plane InGaN/GaN QW systems, we find that the radiative lifetime varies across the PL spectrum noticeably, for the $c$-plane GaN/AlGaN QW system with a very large well width (${L}_{w}=6.8$ nm). However, for system with a narrower width (${L}_{w}=2.4$ nm) the radiative lifetime is approximately constant across the spectrum, even though the decay transitions show a non-exponential behavior. To throw further light onto these findings, we have combined the output of our three-dimensional modified continuum-based model with self-consistent Hartree calculations to take excitonic effects into account. Our calculations reveal exciton localization, with the electron localizing about the hole but still spatially separated along the growth direction due to the macroscopic electrostatic built-in field along the $c$-axis. This is even the case when we include well width fluctuations in the model. Furthermore, our calculated radiative lifetimes for the two experimentally studied QW systems show that indeed, despite these exciton localization effects, the radiative lifetime is affected by alloy fluctuations at the well/barrier interface. Also, we find here that these variations are more pronounced in a wider well when compared to systems with a narrower well width. This finding is consistent with the experimental observation of a much more pronounced variation of the radiative lifetime $\tau$ across the PL spectrum in case of the wider well when compared to the narrower well. Overall, we relate this back to alloy induced variations in the built-in field at the well/barrier interface and connected variations in the electron and hole wave function overlap. The insights gained here, namely about the nature of the recombination, sheds light onto the fundamental properties of GaN/AlGaN QW systems, which is important for driving the development of future state-of-the art UV LEDs forward. The fact that in GaN/AlGaN QWs the carriers are co-localized as excitons, (rather than localized as separate electrons and holes at spatially separated locations) implies that concerns raised about potential negative impacts of localization on the efficiency of green LEDs^55,14^ are not relevant to the performance of UV LEDs. This might imply that efforts to engineer improved localization centres via changes to the quantum well morphology or compositional distribution are unlikely to have unintended detrimental effects.

## Methods

In this section we present and discuss the details of the different applied experimental and theoretical methods. First, the structural characterization techniques applied will be discussed. This is followed by an overview of the techniques employed to study the optical properties of the $c$-plane GaN/AlGaN QWs. Finally, the theoretical framework used in this study is presented.

### Growth and structural characterization

Two 10-period GaN/AlGaN MQW structures were grown by metal-organic chemical vapor deposition in a Thomas Swan 6 $\times$ 2” close-coupled showerhead reactor using trimethylaluminium (TMA), trimethylgallium (TMG) and ammonia (NH${}_{3}$) as precursors and hydrogen as the carrier gas. In-situ 3-wavelength optical monitoring and emissivity-corrected pyrometery were provided by a Laytec EpiTT set-up. Pseudo-substrates consisting of a 5 $\mu$m thick GaN buffer layer were grown first on (0001) $c$-plane sapphire substrates. The growth of the GaN/AlGaN MQW structures on the pseudo-substrates began with a GaN connecting layer of $500$ nm thickness to bury the regrowth interface. The QWs were then grown at 1050 ${}^{\circ }$C and 50 Torr using a TMG flow of 100 $\mu$mol/min giving a GaN growth rate of 0.30 nm/s, while the barriers were grown under the same conditions with the same TMG flow of 100 $\mu$mol/min and a TMA flow of 38 $\mu$mol/min. Hence, the TMA flow was switched to vent or reactor to grow the wells and barriers, respectively, while the ammonia flow was constant at 228 mmol/min throughout the MQW stack growth. The structural properties of both samples were analyzed by high resolution X-ray diffraction (XRD) and transmission electron microscopy (TEM). For the XRD measurements a Philips X’pert diffractometer, equipped with a Cu-K${\alpha }_{1}$ source ($\lambda =1.54056$ Å), an asymmetric 4-crystal Bartels monochromator, and a single point detector was used. Symmetric $\omega$-2$\theta$-scans of the 0002 reflection were performed with an open detector as well as with an additional triple axis monochromator directly in front of the detector. The experimental data were fitted with intensity profiles simulated with the PANalytical Epitaxy software package in order to extract information on the Al-content of the barriers and the thicknesses of QWs and barriers. The samples were observed in cross-section by HAADF-STEM in an FEI Tecnai Osiris microscope operated at 200 kV. The samples were prepared by standard mechanical polishing followed by Ar${}^{+}$ ion milling at 5 kV until a hole formed in the foil and followed by a cleaning at 1 kV down to 0.1 kV.

### Photoluminescence Characterization

The samples were mounted on the cold finger of a closed cycle helium cryostat capable of varying the temperature over the range of 10-300 K to perform either PL spectroscopy or PL time decay measurements. The excitation light for the temperature dependent PL spectroscopy measurements was the mechanically chopped 325 nm (3.81 eV) output of continuous-wave He-Cd laser, which was focused onto the sample with a 15 cm focal length lens. A charge-coupled device beam profiler was used to measure an excitation spot radius of 72 $\mu$m. The samples were both inclined at Brewster’s angle to minimize the distorting effect of Fabry-Pérot interference on the measured PL spectra, with the un-reflected $p$-polarised PL selected by a linear UV polarizer^56^. The collected PL was focused onto the entrance slit of a 0.85 m double grating monochromator with 24 Å resolution and detected with a cooled GaAs photomultiplier tube using standard lock-in techniques. PL decay time measurements were also performed using a mode-locked Ti:sapphire laser outputting 100 fs pulses at a repetition rate of 80 MHz, with a central wavelength of 800 nm and a pulse energy of 1.18  $\times$ 10${}^{-10}$ J. A pulse-selector was used to vary the repetition rate of the pulses and the resulting output was fed to a frequency tripler, wherein pulses with a central wavelength of 266 nm (4.66 eV) were generated from the near-infrared light by second- and third-harmonic generation. The PL time decay measurements were performed using pulses with a repetition rate of 1 MHz for Sample A and 260 kHz for Sample B. A microchannel plate with a time resolution of 80ps was used to detect the emitted light from both samples and standard single-photon counting techniques were used to generate statistics of photon count as a function of time for detection energies chosen across the PL spectra.

### Theoretical Framework

To study the electronic structure of the here considered $c$-plane GaN/AlGaN MQW systems we proceed in the following way. We use a single band effective mass approximation for both electrons and holes. Since we are mainly interested in general trends and alloy induced carrier localization effects, a detailed study of transition energies or absolute values for radiative lifetimes is beyond the scope of the present study. Thus a single band effective mass approach is sufficient for our purpose. Previous three-dimensional modified continuum-based studies on carrier localization effects have used a similar approach^17,21^.

In case of the virtual crystal approximation (VCA), the material parameter set describing the Al${}_{0.18}$Ga${}_{0.82}$N barrier material is obtained by a linear interpolation between the parameter sets of GaN and AlN. Thus each grid point in the barrier material in our three-dimensional simulation box is described by the same averaged material parameter set. Consequently, in VCA there are no spatial variations in the barrier region in terms of the Al content. The relevant material parameters used in the calculations are taken from the literature^53,57,28^. However, it is noteworthy to mention that there exists a large degree of uncertainties in material parameters for AlGaN alloys especially in (band gap) bowing parameters^28,58^ and band offset values^27^. Therefore, absolute values of the different output parameters such as transition energies and radiative lifetime will depend on accurate knowledge of material parameters and also their composition dependence^59^. But, as already stressed above, we are interested in general trends rather than providing a one-to-one theory experiment comparison on transition energies or radiative lifetime values.

Going beyond the VCA, spatial variations in Al content are treated as follows. In the barrier region, randomly 18% of the grid points have been described by AlN parameters and the remaining 82% by a GaN parameter set. In doing so, the Al content fluctuates throughout the barrier material and different configurations are realized, but at the same time, on average, the Al content in the barrier is 18%.

Overall, our single-band effective mass treatment, along with the VCA and random alloy treatment, is implemented in the S/Phi/nX software library^60,61^. Details of the implementation and the general features of the package are described elsewhere^62^. Details on our self-consistent Hartree approach are also given in the literature^53^. From these calculations we obtain exciton energies (${E}_{X}$) and oscillator strength/wave function overlaps ($f$) in the presence of the attractive Coulomb interaction between electron and hole. Equipped with this knowledge, the radiative lifetime ${\tau }^{theo}$ is calculated via^63^

1 $${\tau }^{theo}=\frac{2\pi {\varepsilon }_{0}{m}_{0}{c}^{3}{\hslash }^{2}}{n{e}^{2}{({E}^{X})}^{2}f}\ \ ,$$

where $n$, ${\varepsilon }_{0}$, ${m}_{0}$, $c$, $\hslash$ denote the barrier refractive index, vacuum permittivity, the free electron mass, the vacuum speed of light and the reduced Planck’s constant.

Regarding the numerical aspects of our approach, for the narrower well-width (${L}_{w}=2.5$ nm) all calculations have been performed on a $40\times 40\times 12$ nm${}^{3}$ supercell with periodic boundary conditions. For the wider well (${L}_{w}=6.75$ nm), the supercell size is chosen to be $40\times 40\times 17$ nm${}^{3}$, again with periodic boundary conditions. This slightly larger simulation box has been chosen to keep the barrier width, accounting for periodic boundary conditions, at approximate 10 nm. Our results presented in the main text are evaluated with a step size of 0.25 nm and 0.5 nm along the growth and in-plane directions, respectively. Due to this choice of step size, the well-width considered in our calculations are ${L}_{w}=2.5$ nm and ${L}_{w}=6.75$ nm, respectively, which is very close to the experimental values. The grid size along the growth direction has been chosen, based on an ideal GaN wurtzite structure, so that approximately one Ga-N bond would be included, motivating the use of pure GaN or AlN parameters at each lattice site. The in-plane step size of 0.5 nm together with the out-of plane step size ensures then that, when looking at this from a microscopic wurtzite picture, at least one Ga-N-Ga chain would approximately fall in this volume. For InGaN alloys it has been shown that a chain of In-N-In embedded in GaN is sufficient to lead to carrier localization effects^12^. This idea is here carried over to Ga-N-Ga chains embedded in AlN. Nevertheless, we have also tested (not shown here) smaller step sizes to analyze the robustness of the results against changes in these parameters. More specifically, a finer grid size of 0.1 nm and 0.25 nm along the growth and in-plane direction, respectively, have been used. With these setting qualitatively the same results as discussed in the main text of the manuscript have been obtained. Based on all this, we have carried out our calculations with a step size of 0.25 nm and 0.5 nm along the growth and in-plane directions, respectively.

## References

[1] Peltola SM, Melchels FPW, Grijpma DW, Kellomaki M (2008). A review of rapid prototyping techniques for tissue engineering purposes. Ann. Med..

[2] Schreiner M, Martinez-Abaigar J, Glaab J, Jansen M (2014). UV-B induced secondary plant metabolites. Optik Photonik.

[3] Hargis, P. J. *et al*. Ultraviolet fluorescence identification of protein, DNA, and bacteria (1995).

[4] Mellqvist J, Rosén A (1996). Doas for flue gas monitoring. temperature effects in the UV/visible absorption spectra of NO, NO_2, SO_2 and NH_3. J. Quant. Spectrosc. Radiat. Transf.

[5] Lee, J., Hong, H., Kim, K. & Park, K. A survey on banknote recognition methods by various sensors. *Sensors* **17** (2017). 10.3390/s17020313 PMC5335928 28208733

[6] Leghissa A, Smuts J, Qiu C, Hildenbrand Z, Schug K (2017). Detection of cannabinoids and cannabinoid metabolites using gas chromatography with vacuum ultraviolet spectroscopy. Sep. Sci. Plus.

[7] Bi H (2016). Performance enhanced UV/Vis spectroscopic microfluidic sensor for ascorbic acid quantification in human blood. Biosens. Bioelectron.

[8] Shklyaev, A. A., Gorbunov, A. V. & Ichikawa, M. Excitation-dependent blue shift of photoluminescence peak in 1.5–1.6 {\it{μ}}m wavelength region from dislocation-rich Si layers. In *2010 11th International Conference and Seminar on Micro/Nanotechnologies and Electron Devices*, 59–63 (2010).

[9] Cho Y-H (1998). ‘S-shaped’ temperature-dependent emission shift and carrier dynamics in InGaN/GaN multiple quantum wells. Appl. Phys. Lett..

[10] Chichibu S, Wada K, Nakamura S (1997). Spatially resolved cathodoluminescence spectra of InGaN quantum wells. Appl. Phys. Lett..

[11] Bai J, Wang T, Sakai S (2000). Influence of the quantum-well thickness on the radiative recombination of InGaN/GaN quantum well structures. Appl. Phys. Lett..

[12] Chichibu, S. F. *et al*. Origin of defect-insensitive emission probability in In-containing (Al,In,Ga)N alloy semiconductors. *Nature Mater.* **5**, 810 (2006). 10.1038/nmat1726 16951678

[13] Dawson P, Schulz S, Oliver RA, Kappers MJ, Humphreys CJ (2016). The nature of carrier localization in polar and nonpolar InGaN/GaN quantum wells. J. Appl. Phys..

[14] Jeong H (2015). Carrier localization in In-rich InGaN/GaN multiple quantum wells for green light-emitting diodes. Sci. Rep..

[15] Kim M (2017). Investigating carrier localization and transfer in InGaN/GaN quantum wells with V-pits using near-field scanning optical microscopy and correlation analysis. Sci. Rep..

[16] Karpov SY (2017). Carrier localization in InGaN by composition fluctuations: implication to the “green gap”. Photon. Res..

[17] Rigutti L (2016). Statistical nanoscale study of localised radiative transitions in GaN/AlGaN quantum wells and epitaxial layers. Semicond. Sci. Technol..

[18] Gallart M (2000). Scale effects on exciton localization and nonradiative processes in GaN/AlGaN quantum wells. Phys. Status Sol A.

[19] Yu H, Lee LK, Jung T, Ku PC (2007). Photoluminescence study of semipolar InGaN-GaN multiple quantum wells grown by selective area epitaxy. Appl. Phys. Lett..

[20] Li Q, Xu SJ, Xie MH, Tong SY (2005). Origin of the ‘S-shaped’ temperature dependence of luminescent peaks from semiconductors. J. Phys-Condens. Mat..

[21] Watson-Parris D (2011). Carrier localization mechanisms in In_*x*Ga_1-*x*N/GaN. Phys. Rev. B.

[22] Rubel O (2005). Quantitative description of disorder parameters in (GaIn)(NAs) quantum wells from the temperature-dependent photoluminescence spectroscopy. J. Appl. Phys..

[23] Pecharromán-Gallego R, Martin RW, Watson IM (2004). Investigation of the unusual temperature dependence of InGaN/GaN quantum well photoluminescence over a range of emission energies. J. Phys. D: Appl. Phys.

[24] Monemar B (2003). Influence of polarization fields and depletion fields on photoluminescence of AlGaN/GaN multiple quantum well structures. Phys. Status Solidi B.

[25] Haratizadeh H (2007). Optical observation of discrete well width fluctuations in wide band gap III-nitride quantum wells. Phys. Status Solidi B.

[26] Williams DP, Schulz S, Andreev AD, O’Reilly EP (2009). Theory of GaN quantum dots for optical applications. IEEE J. Sel. Topics. Quantum Electron..

[27] Moses PG, Miao M, Yan Q, Van de Walle CG (2011). Hybrid functional investigations of band gaps and band alignments for AlN, GaN, InN, and InGaN. J. Chem. Phys..

[28] Coughlan C, Schulz S, Caro MA, O’Reilly EP (2015). Band gap bowing and optical polarization switching in AlGaN alloys. Phys. Status Solidi B.

[29] Caro MA, Schulz S, O’Reilly EP (2013). Theory of local electric polarization and its relation to internal strain: Impact on polarization potential and electronic properties of group-III nitrides. Phys. Rev. B.

[30] Chuang SL, Chang CS (1996). k · p method for strained wurtzite semiconductors. Phys. Rev. B.

[31] Jeon N (2015). Alloy fluctuations act as quantum dot-like emitters in GaAs-AlGaAs core-shell nanowires. ACS Nano.

[32] Valcheva E (2007). Influence of well-width fluctuations on the electronic structure of GaN/AlGaN multiquantum wells with graded interfaces. Acta Physica Polonica A.

[33] Park S-H, Chuang S-L (2000). Spontaneous polarization effects in wurtzite GaN/AlGaN quantum wells and comparison with experiment. Appl. Phys. Lett..

[34] Massabuau FC-P (2015). Investigation of unintentional indium incorporation into GaN barriers of InGaN/GaN quantum well structures. Phys. Status Solidi B.

[35] Oliver R (2010). Microstructural origins of localization in InGaN quantum wells. J. Phys. D: Appl. Phys.

[36] Dingle R, Ilegems M (1971). Donor-acceptor pair recombination in GaN. Solid State Commun.

[37] Reshchikov MA, Morkoc H (2005). Luminescence properties of defects in GaN. J. Appl. Phys..

[38] Reshchikov MA, Yi G-C, Wessels BW (1999). Behavior of 2.8- and 3.2-ev photoluminescence bands in Mg-doped GaN at different temperatures and excitation densities. Phys. Rev. B.

[39] Fischer S, Wetzel C, Haller EE, Meyer BK (1995). On p-type doping in GaN acceptor binding energies. Appl. Phys. Lett..

[40] Moore WJ, Freitas JA, Lee SK, Park SS, Han JY (2002). Magneto-optical studies of free-standing hydride-vapor-phase epitaxial GaN. Phys. Rev. B.

[41] Schulz S, Caro MA, Coughlan C, O’Reilly EP (2015). Atomistic anaylsis of the impact of alloy and well width fluctuations on the electronic and optical properties of InGaN/GaN quantum wells. Phys. Rev. B.

[42] Varshni Y (1967). Temperature dependence of the energy gap in semiconductors. Physica.

[43] Vurgaftman I, Meyer JR (2003). Band parameters for nitrogen-containing semiconductors. J. Appl. Phys..

[44] Bell A (2004). Exciton freeze-out and thermally activated relaxation at local potential fluctuations in thick AlGaN layers. J. Appl. Phys..

[45] Leroux M (1998). Quantum confined stark effect due to built-in internal polarization fields in (Al,Ga)N/GaN quantum wells. Phys. Rev. B.

[46] Sabooni M (2007). Exciton localization behaviour in different well width undoped GaN/Al_0.07Ga_0.93N nanostructures. Opto-Electron. Rev..

[47] Morel A (2003). Donor-acceptor-like behavior of electron-hole pair recombinations in low-dimensional (Ga,In)N/GaN systems. Phys. Rev. B.

[48] Davidson JA (2000). Photoluminescence studies of InGaN/GaN multi-quantum wells. Semicond. Sci. Technol..

[49] Davies MJ (2016). A comparison of the optical properties of ingan/gan multiple quantum well structures grown with and without si-doped ingan prelayers. J. Appl. Phys..

[50] Sousa MA (2015). Luminescence studies on green emitting ingan/gan mqws implanted with nitrogen. Sci. Rep..

[51] Lefebvre P (1998). Recombination dynamics of free and localized excitons in GaN/Ga0.93Al0.07N quantum wells. Phys. Rev. B.

[52] Tanner DSP, McMahon JM, Schulz S (2018). Interface roughness, carrier localization, and wave function overlap in c-plane (In,Ga)N/GaN quantum wells: Interplay of well width, alloy microstructure, structural inhomogeneities, and coulomb effects. Phys. Rev. Appl.

[53] Patra SK (2017). Theoretical and experimental analysis of radiative recombination lifetimes in nonpolar InGaN/GaN quantum dots. Phys. Status Solidi B.

[54] Schulz S (2015). Structural, electronic, and optical properties of m-plane InGaN/GaN quantum wells: Insights from experiment and atomistic theory. Phys. Rev. B.

[55] Auf der Maur M, Pecchia A, Penazzi G, Rodrigues W, Di Carlo A (2016). Unraveling the green gap" problem: The role of random alloy fluctuations in InGaN/GaN light emitting diodes. Phys. Rev. Lett..

[56] Graham DM (2005). Optical and microstructural studies of InGaN/GaN single-quantum-well structures. J. Appl. Phys..

[57] Rinke P (2008). Consistent set of band parameters for the group-III nitrides AlN, GaN, and InN. Phys. Rev. B.

[58] Neuschl B (2014). Composition dependent valence band order in c-oriented wurtzite AlGaN layers. J. Appl. Phys..

[59] Patra SK, Marquardt O, Schulz S (2016). Polar, semi- and non-polar nitride-based quantum dots: influence of substrate orientation and material parameter sets on electronic and optical properties. Opt. Quant. Electron..

[60] Marquardt O (2014). A generalized plane-wave formulation of formalism and continuum-elasticity approach to elastic and electronic properties of semiconductor nanostructures. Comput. Mater. Sci..

[61] Boeck S, Freysoldt C, Dick A, Ismer L, Neugebauer J (2011). The object-oriented DFT program library S/PHI/nX. Comput. Phys. Commun..

[62] Patra SK, Schulz S (2017). Non-polar InxGa1-xN/GaN quantum dots: impact of dot size and shape anisotropies on excitonic and biexcitonic properties. J. Phys. D: Appl. Phys.

[63] Fonoberov VA, Balandin AA (2003). Excitonic properties of strained wurtzite and zinc-blende GaN/Al_*x*Ga_1-*x*N quantum dots. J. Appl. Phys..

