# Publisher Correction: Impact of alloy fluctuations and Coulomb effects on the electronic and optical properties of c-plane GaN/AlGaN quantum wells

**DOI:** 10.1038/s41598-020-62494-x

**Published:** 2020-03-24

**Authors:** A. A. Roble, S. K. Patra, F. Massabuau, M. Frentrup, M. A. Leontiadou, P. Dawson, M. J. Kappers, R. A. Oliver, D. M. Graham, S. Schulz

**Affiliations:** 1 Department of Physics and Astronomy, and Photon Science Institute, The University of Manchester, Manchester, M13 9PL United Kingdom; 2 Tyndall National Institute, University College Cork, Cork, T12 R5CP Ireland; 3 Department of Electrical Engineering, University College Cork, Cork, T12 YN60 Ireland; 4 Department of Materials Science and Metallurgy, University of Cambridge, 27 Charles Babbage Road, Cambridge, CB3 0FS United Kingdom; 5 Present Address: School of Science, Engineering and Environment, University of Salford, Salford, Greater Manchester M5 4WT United Kingdom

Correction to: *Scientific Reports* 10.1038/s41598-019-53693-2, published online 11 December 2019

This Article contains a typographical error in the Results and Discussion section under subheading ‘Experimental analysis of the optical properties’ where,

“{L}_{w}  =  2.4 nm”

should read:

“L_w_ = 2.4 nm”

